# Open Innovation in Medical and Pharmaceutical Research: A Literature Landscape Analysis

**DOI:** 10.3389/fphar.2020.587526

**Published:** 2021-01-14

**Authors:** Andy Wai Kan Yeung, Atanas G. Atanasov, Helen Sheridan, Elisabeth Klager, Fabian Eibensteiner, Sabine Völkl-Kernsock, Maria Kletecka-Pulker, Harald Willschke, Eva Schaden

**Affiliations:** ^1^ Oral and Maxillofacial Radiology, Applied Oral Sciences and Community Dental Care, Faculty of Dentistry, The University of Hong Kong, Hong Kong, China; ^2^ Ludwig Boltzmann Institute for Digital Health and Patient Safety, Medical University of Vienna, Vienna, Austria; ^3^ Institute of Genetics and Animal Biotechnology of the Polish Academy of Sciences, Magdalenka, Poland; ^4^ Institute of Neurobiology, Bulgarian Academy of Sciences, Sofia, Bulgaria; ^5^ Department of Pharmacognosy, University of Vienna, Vienna, Austria; ^6^ NatPro Centre. School of Pharmacy and Pharmaceutical Sciences, Trinity College Dublin, Dublin, Ireland; ^7^ Division of Pediatric Nephrology and Gastroenterology, Department of Pediatrics and Adolescent Medicine, Comprehensive Center for Pediatrics, Medical University of Vienna, Vienna, Austria; ^8^ Department of Anaesthesia, Intensive Care Medicine and Pain Medicine, Medical University Vienna, Vienna, Austria

**Keywords:** public-private partnership, pharmaceutical industry, drug discovery, patient-centered innovation, intellectual property, crowdsourcing, knowledge management, open innovation

## Abstract

Open innovation in medical and pharmaceutical research has grown steadily over the last decade. However, the performance of the published literature in terms of the scientific impact and gaining social media attention remains largely unexplored. The scientific literature of open innovation was examined by means of bibliometric analyses to identify the most prolific authors, organizations, countries, journals, research areas, and recurring terms. By accessing the Web of Science Core Collection and Altmetric electronic databases, citation-related and Altmetric data were evaluated. Public-private partnerships and a selection of newly introduced potential novel drugs in the analyzed publications were identified. North America and Europe were the major literature contributors. Research outputs were mainly published in journals focused on business and economics, pharmacology and pharmacy, and engineering. Many pharmaceutical and biotechnological companies contributed to the analyzed publications, with higher mean citation counts and social media attention (Altmetric score) than nonindustry articles. Public-private partnerships fostered financial support, sharing of expertise and intellectual property, and research collaborations. In summary, open innovation might serve as a powerful strategy to both benefit the involved industry entities and accelerate the development of solutions and products for the betterment of human health.

## Introduction

Companies rely on innovation in order to develop and stay competitive in the face of dynamically evolving scientific, technological, and societal conditions. Traditionally, the industry's approach has been to generate innovation internally within specialized research and development departments. However, the shortcomings of this approach are that, on the one hand, there is always a lot of potentially valuable innovation generated in parallel outside of companies and, on the other hand, internally generated innovation cannot always be translated into commercial products or services benefiting the company. Therefore, in recent times, there has been a paradigm shift toward open innovation, a model that does not exclude the traditional internal innovation but is open to also incorporate externally generated innovation (Hodson, 2016). Open innovation, as defined by Chesbrough and Bogers (2014), is “a distributed innovation process based on purposively managed knowledge flows across organizational boundaries, using pecuniary and nonpecuniary mechanisms in line with the organization's business model.” The innovation derived from external sources can include ideas, technologies, and research and development. In open innovation, companies have moved from the pursuit of intellectual property protection to engaging in cross industry-academia and industry-public collaboration and engagement. Early adopters of the open innovation concept have been the so-called “high-technology” industries in the area of information technology, computers, and pharmaceuticals. Beyond these early adopters, other industrial areas with medical significance that shortly followed embracing the open innovation concept include industries from the areas of medical devices, chemicals, and bioscience tools and services (Chesbrough and Crowther, 2006). Depending on the direction of movement of new knowledge, three different types of open innovation processes have been distinguished (Gassmann and Enkel, 2004). The outside-in (inbound) process involves the integration of internal knowledge of an organization with knowledge derived from external sources; the inside-out (outbound) process involves generating profits for the organization by transferring internally generated knowledge or intellectual property to external entities; and the third, “coupled,” process involves a combination of both inbound and outbound processes by the formation of alliances with complementary external entities (Gassmann and Enkel, 2004). Analysis of the literature on open innovation in healthcare revealed that open inbound innovation was the most widely applied approach in this sector (Wass and Vimarlund, 2016).

In pharmaceutical companies, open innovation differs according to partnerships, incentives, and goals (Bianchi et al., 2011). Schuhmacher et al. (2013) placed open innovation models into four categories, the knowledge creator, knowledge translator, knowledge integrator, and knowledge leverager, and assigned pharmaceutical companies to these models, depending on their specific approach (Schuhmacher et al., 2013). As a prominent example from the pharma industry, Eli Lilly has a strong history of collaboration that led to launching of their Open Innovation Drug Discovery program in 2009 (Alvim-Gaston et al., 2014).

To foster academia-industry interactions, a significant number of universities have created formal Academic Drug Discovery Centres (ADDCs), which include industrial collaboration (Harris et al., 2015). One of the earliest centers, the Actar AB center, was created at the Karolinska Institute in Sweden in 2000, followed by the Harvard NeuroDiscovery center in the US in 2001, with dozens of more ADDCs established in the EU and the US in the following decade (Kirkegaard and Valentin, 2014). Building on the ADDC model, another concept that has emerged in the academia-industry interface is that of the Academic Drug Discovery Consortium. One of the first consortia was founded in the EU, and the longest existing academia-industry collaboration was the formation of the Division of Signal Transduction Therapy between the University of Dundee, six pharma companies, and twenty academic research teams (http://www.lifesci.dundee.ac.uk/unit/division-signal-transduction-therapy-dstt). This type of collaborative initiative can extend beyond the sharing of costs and may improve the efficiency of drug development and reduce duplication of effort (Savage, 2016). Regulators and governments are also increasingly engaged in supporting drug discovery, e.g., in Sweden where the government ring-fenced early-stage funding of €6 M per year (2013–2016) for the Science for Life Drug Discovery and Development platform (SciLifeLab DDD) established in 2011 (Arvidsson et al., 2016). Further examples of regulator and government engagement are the Critical Path Initiative in the United States (FDA) (Woodcock and Woosley, 2008) and the Cooksey Review of health research funding in the United Kingdom (Black, 2006). With the advancement of Internet technology, collaborative work from a distance gained new significance and was manifested in a variety of different forms potentially benefiting open innovation, including the formation of virtual organizations, virtual enterprises, and collaborative virtual laboratories (Camarinha-Matos and Afsarmanesh, 2005; Camarinha-Matos et al., 2006).

Crowdsourcing represents an open innovation approach gaining momentum, which can be used to derive solutions that advance the medical field by collective intelligence and resources and actively engaging patients and the general public. Crowdsourcing approaches used in the context of medical research include innovation challenges (e.g., prize contests), hackathons (short events usually bringing participants together for a few days around a common theme with the aim of solving defined challenges, often with a focus on the area of computer programming), and online systems for collaboration (e.g., websites supporting the engagement of visitors to solve a defined problem) (Tucker et al., 2019).

The direct involvement of nonprofessionals in scientific research, referred to as “citizen science,” is a highly potent collaborative approach for gaining new knowledge (Schnoor, 2007; Bonney et al., 2009). Citizen science has been well-established in research fields, such as ecology and astronomy, and in the context of increasing significance of patient-centered medicine. Currently, there are broad ongoing efforts for citizen science implementation in health and biomedical research, whereby progress in these areas encounters some difficulties related to existing ethical issues, including consideration for possible risks for the involved citizen scientists or other humans (Wiggins and Wilbanks, 2019).

Our goal in this work is to gain new insights into open innovation in medical and pharmaceutical research by performing a full-scale bibliometric analysis, an approach that has proven its value in the quantitative characterization of various outputs of the scientific literature published in other biomedical research areas (Yeung et al., 2018; Yeung et al., 2019a; Yeung et al., 2019b; Cen et al., 2020; Chen and Wang, 2020; Fortuna et al., 2020). Such full-scale bibliometric analysis might reveal information for major institutions, top scientists of the area, research impacts (reflected by citation counts), and main research topics and trends. Researchers and nonexperts can use such data for rapid orientation in the respective scientific area and to achieve some more specific goals, for example, identifying potential new collaboration partners. Open innovation, in general, as well as in medical research in particular, is currently a major trend with vibrant activities apart from publishing research, such as the organization of hackathons (Angelidis et al., 2016) and the establishment of innovation hubs (Rifai et al., 2015). In order to better understand participants and publishing trends in this research area, the present work focuses on the bibliometric characterization of its publication landscape.

## Materials and Methods

The digital literature database, Web of Science Core Collection, was accessed in early June 2020 in order to identify and analyze relevant publications. We therefore utilized the following search string: TS = (“open innovation*” OR “external innovation*” OR “patient cent* innovation*”) AND TS = (medic* OR illness* OR disease* OR health* OR pharma*). This search strategy recognized publications presenting the indicated phrases or their derivatives in the title, abstract, or keyword fields. The bibliographic information of the yielded publications was analyzed to reveal the most productive entities in terms of authors, organizations, countries, journals, research areas, etc. The complete records were exported to VOSviewer (www.vosviewer.com, version 1.6.15, Leiden University, The Netherlands), a dedicated bibliometric software, for further analyses and visualization of bibliometric networks. As a subanalysis, crowdsourc* was searched within the dataset in order to identify relevant articles.

Nonparametric tests were used as the data were not normally distributed as tested by Shapiro–Wilk tests. Mann–Whitney *U* tests were conducted to test if publications with contributions from the industry (i.e., authors affiliated with biotechnology/pharmaceutical companies) had higher mean citation counts per publication than their nonindustry counterparts. Similarly, the group difference in Altmetric Attention Score, a score indicating social media attention tracked by Altmetric (www.altmetric.com), was tested. Articles were considered to have industrial contributions when at least one of the authors was affiliated with industry (articles published jointly with industry professionals and academic researchers were considered to have industrial contribution).

## Results

**FIGURE 1:**
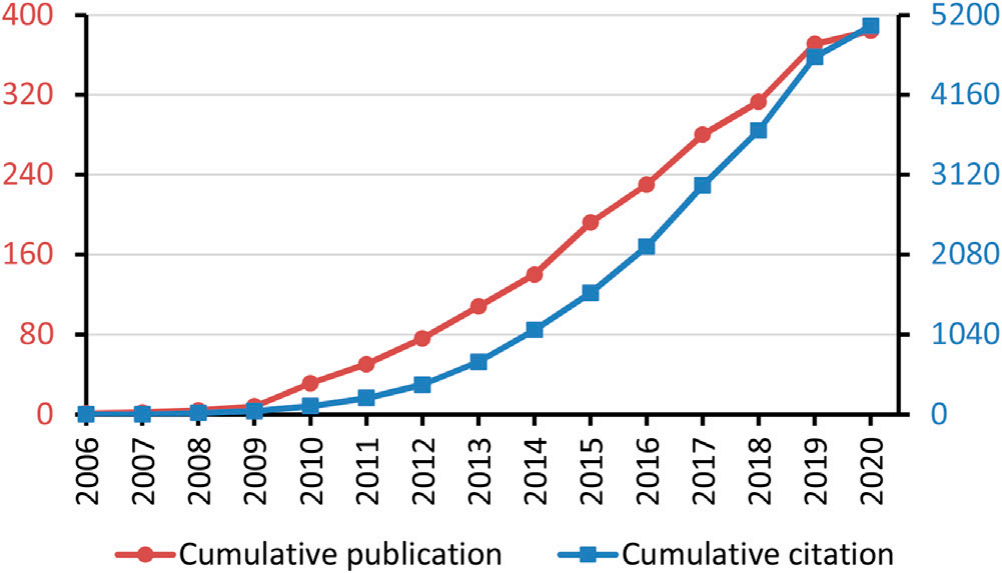
Cumulative publication and citation counts of open innovation in medical and pharmaceutical research.

The search yielded 384 publications that accumulated over 5,000 citations (Figure 1), with an h-index of 32 and 13.2 citations per publication (CPP). There were 240 articles and 55 review publications, equivalent to a ratio of 4.4–1. There were 54 proceedings and 27 editorial materials. The first article was published in 2006, which used Millenium Pharmaceuticals (now known as Takeda Oncology, a subsidiary of Takeda Pharmaceutical) as one of the examples of early adopters of open innovation in the high-technology industries (Chesbrough and Crowther, 2006).

The 10 most prolific authors, organizations (additionally including companies in the industry with two or more articles), countries, journals, and research areas are listed in Table 1. Most of the top 10 most prolific authors came from Italy, in particular from the University of Salerno. Four of the top 10 most prolific organizations were pharmaceutical companies. The top 10 countries were situated in North America and Europe, except China. Nine of the top 10 journals had an impact factor and were mostly concerning business/economics and pharmacology/pharmacy.

**TABLE 1:** The 10 most prolific authors, organizations (additionally including companies in the industry with two or more articles), countries, journals, and research areas.

	No. of publications (% of 384)	Citations per publication
*Authors*		
Antonello Cammarano	16 (4.2)	8.6
Mauro Caputo	16 (4.2)	8.6
Francesca Michelino	16 (4.2)	8.6
Emilia Lamberti	14 (3.6)	9.9
I. Sam Saguy	5 (1.3)	18.6
Alexander Schuhmacher	5 (1.3)	27.8
Giustina Secundo	5 (1.3)	4.6
Federico Frattini	4 (1.0)	63.8
Oliver Gassmann	4 (1.0)	37.8
Giuseppina Passiante	4 (1.0)	3.0
*Organizations*		
University of Salerno	18 (4.7)	7.7
University of London	10 (2.6)	14.1
AstraZeneca (industry)	8 (2.1)	44.9
Eli Lilly (industry)	8 (2.1)	15.4
GlaxoSmithKline (industry)	8 (2.1)	48.0
University of Rome Tor Vergata	8 (2.1)	6.8
Hebrew University of Jerusalem	6 (1.6)	16.3
Novartis (industry)	6 (1.6)	30.7
University of California system	6 (1.6)	137.2
Vrije Universiteit Amsterdam	6 (1.6)	27.0
Bayer AG (industry)	5 (1.3)	30.6
Johnson & Johnson (industry)	4 (1.0)	72.0
Sanofi S.A. (industry)	4 (1.0)	7.5
Boehringer Ingelheim (industry)	3 (0.8)	7.7
Pfizer (industry)	3 (0.8)	29.7
*Countries*		
United States	95 (24.7)	26.2
United Kingdom	62 (16.1)	15.0
Italy	53 (13.8)	13.0
Germany	42 (10.9)	15.3
Netherlands	28 (7.3)	25.3
Sweden	23 (6.0)	21.1
Spain	20 (5.2)	9.1
Switzerland	20 (5.2)	25.0
Canada	18 (4.7)	8.4
China	18 (4.7)	2.8
*Journals (impact factor)*		
Drug Discovery Today (6.880)	17 (4.4)	24.2
Expert Opinion on Drug Discovery (4.421)	12 (3.1)	9.4
Sustainability (2.592)	10 (2.6)	1.9
International Journal of Innovation Management (NA)	9 (2.3)	6.6
International Journal of Technology Management (1.160)	9 (2.3)	7.1
R&D Management (2.354)	9 (2.3)	91.6
Future Medicinal Chemistry (3.617)	7 (1.8)	4.6
European Journal of Innovation Management (1.793)	6 (1.6)	12.8
Technology Analysis & Strategic Management (1.739)	6 (1.6)	7.0
Research-Technology Management (1.407)	5 (1.3)	10.6
*Research areas* ^a^		
Business and economics	146 (38.0)	17.6
Pharmacology and pharmacy	80 (20.8)	13.0
Engineering	62 (16.1)	16.8
Science technology other topics	28 (7.3)	6.3
Healthcare sciences services	21 (5.5)	6.3
Operations research management science	21 (5.5)	25.2
Computer science	20 (5.2)	5.5
Environmental sciences and ecology	17 (4.4)	4.8
Information science and library science	13 (3.4)	6.7
Food science technology	11 (2.9)	20.7

^a^ Since the search string included the metaword pharma*, readers should be aware that the research area of “pharmacology and pharmacy” would be favored.

There were 51 publications with authors affiliated with biotechnology/pharmaceutical companies, with a mean citation count per publication (CPP) of 23.3 (SD = 39.3), which was significantly higher than purely academic publications (11.6, SD = 43.5) (*p* = 0.001). Similarly, the former group of publications had an Altmetric score of 14.2 (SD = 34.7), significantly higher than the latter group (5.9, SD = 15.7) (*p* = 0.013). The public-private partnerships or initiatives introduced in these 51 industry articles are listed in Table 2.

**TABLE 2:** Public-private partnerships or initiatives introduced in the industry articles concerning open innovation in medical and pharma research.

Project name	Objective	Involved companies
Open PHACTS (Williams et al., 2012)	To deliver and sustain an open pharmacological webspace to facilitate drug discovery research	AstraZeneca
INVITE GmbH (Bieringer et al., 2013)	To innovate, develop, and revolutionize technologies for chemical, pharmaceutical, and biotechnology purposes	Bayer AG
Grants4Targets (Lessl et al., 2011)	To provide financial support and expertise on target validation and drug discovery to external groups	Bayer AG
Phenotypic Drug Discovery (PD2) Initiative (Lee et al., 2011)	To make their phenotypic assays available to external research groups	Eli Lilly
Open Innovation Drug Discovery (OIDD) (Alvim-Gaston et al., 2014; Lee et al., 2015; Carroll et al., 2017)	To identify novel molecules active in relevant disease biology models	Eli Lilly
Africa NCD Open Lab (Hall et al., 2015)	To share expertise and resources to conduct research on noncommunicable diseases in Africa	GlaxoSmithKline
Observational Medical Outcomes Partnership (Stang et al., 2010)	To advance the active surveillance of medical product safety by using existing observational databases	GlaxoSmithKline and Johnson & Johnson
tranSMART (Perakslis et al., 2010)	To maintain a data warehouse for data sharing and partnership	Johnson & Johnson
Sanofi-MIT Partnership (Stewart et al., 2016)	To provide seed funding to academia for pharmaceutical research	Sanofi

**FIGURE 2:**
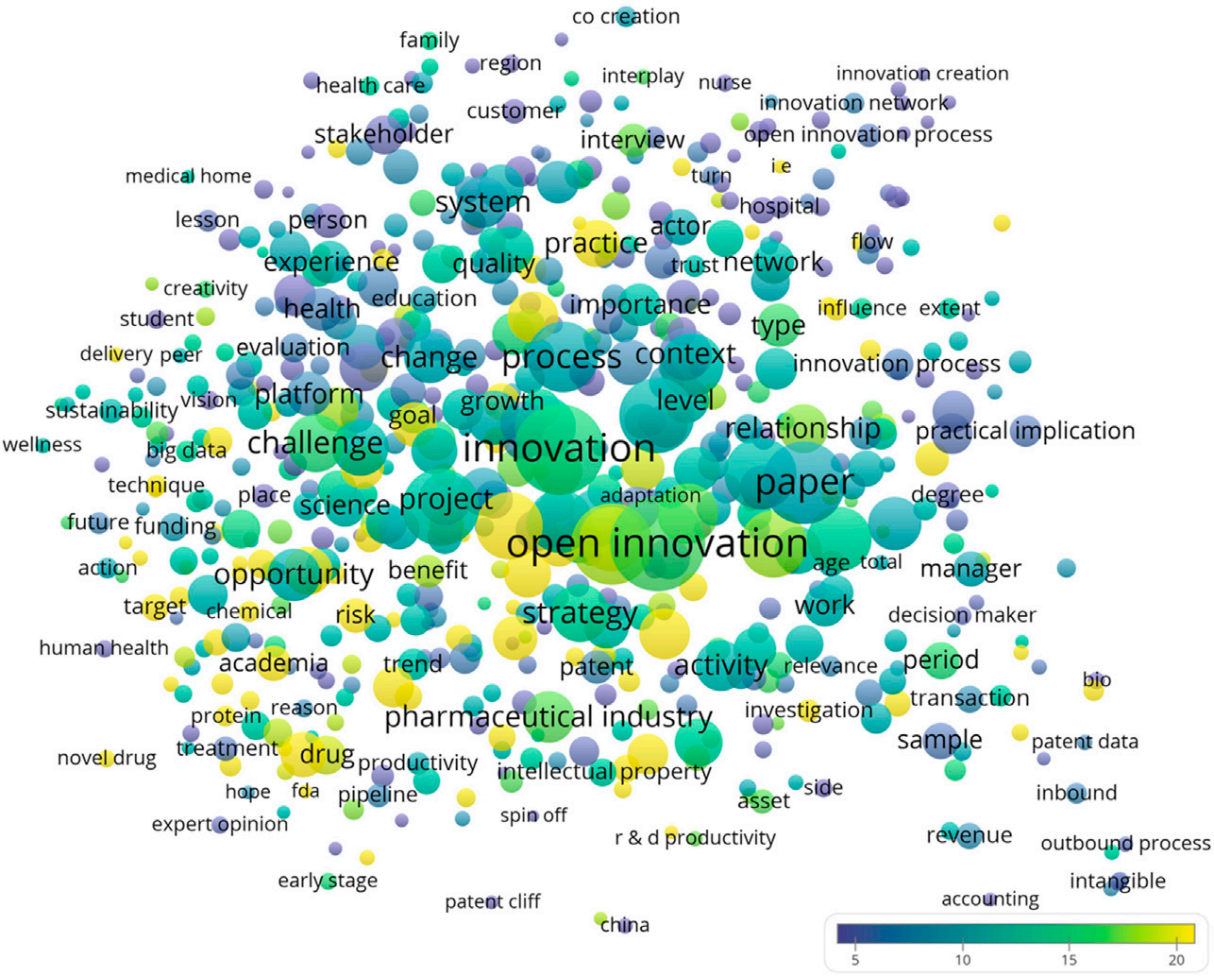
The recurring words and phrases in the titles and abstracts of open innovation in medical and pharmaceutical research publications. Words and phrases recurring in at least 1% of all publications (*n* = 4) were illustrated. The color of the circles represented the citations per publication (CPP) of publications that contained the concerning word or phrase. The size of the circles represented the number of publications. The proximity between the circles represented the frequency of cooccurrence of the concerning words or phrases.

The term map of titles and abstracts showed that academia and pharmaceutical industry collaborated for various aspects, such as novel drug discovery (*n* = 6, CPP = 19.7) and delivery (*n* = 5, CPP = 23.0) (Figure 2). Highly cited terms revealed from the bibliometric analysis were high technology (*n* = 4, CPP = 185.3), outsourcing (*n* = 8, CPP = 94.3), drug administration (*n* = 5, CPP = 67.8), outbound open innovation (*n* = 5, CPP = 50.2), and public-private partnership (*n* = 14, CPP = 34.4) (Table 3). Meanwhile, the top 20 most recurring author keywords are listed in Table 4. Drug discovery, intellectual property, knowledge management, licensing, drug development, and entrepreneurship were among the most highly cited keywords.

**TABLE 3:** Top 20 most recurring and most highly cited terms in the titles and abstracts, identified with VOSviewer. Terms recurring in at least 1% of publications (*n* = 4) were analyzed.

Most recurring	*N* (% of 384)	CPP	Most highly cited	*N* (% of 384)	CPP
Open innovation	184 (47.9)	15.9	High technology	4 (1.0)	185.3
Innovation	168 (43.8)	14.8	Wide range	8 (2.1)	102.5
Paper	139 (36.2)	9.7	Outsourcing	8 (2.1)	94.3
Research	136 (35.4)	17.9	Drug administration	5 (1.3)	67.8
Development	121 (31.5)	12.7	FDA	4 (1.0)	64.8
Study	116 (30.2)	11.1	Paradigm	16	57.9
Analysis	106 (27.6)	11.7	Outbound open innovation	5 (1.3)	50.2
Process	103 (26.8)	10.2	Supply	5 (1.3)	40.8
Model	92 (24.0)	24.6	Medium sized enterprise	7 (1.8)	35.9
Company	92 (24.0)	17.6	Development process	10 (2.6)	35.4
Industry	85 (22.1)	20.4	Mode	10 (2.6)	35.4
Firm	84 (21.9)	14.0	Growth	28 (7.3)	34.8
Approach	82 (21.4)	12.2	Open innovation research	6 (1.6)	34.7
Knowledge	82 (21.4)	11.5	Communication technology	5 (1.3)	34.6
Technology	76 (19.8)	12.6	Public private partnership	14 (3.6)	34.4
Data	75 (19.5)	16.6	NPD	6 (1.6)	34.3
Collaboration	70 (18.2)	11.2	Pharmaceutical firm	14 (3.6)	33.9
Challenge	69 (18.0)	15.3	Practice	46 (12.0)	32.1
Strategy	69 (18.0)	14.0	Bio-pharmaceutical industry	9 (2.3)	31.9
Need	65 (16.9)	11.6	Success factor	6 (1.6)	31.8

CPP, citations per publication; FDA, United States Food and Drug Administration; NPD, new product development.

**TABLE 4:** Top 20 most recurring author keywords.

Author keyword	*N* (out of 384)	Citations per publication
Open innovation	159	9.7
Innovation	33	8.8
Pharmaceutical industry	23	4.5
Drug discovery	17	10.6
Collaboration	12	4.3
Intellectual property	12	17.5
Crowdsourcing	11	5.5
Knowledge management	11	16.6
Bio-pharmaceutical	10	4.3
Healthcare	8	7.6
Innovation management	8	5.6
Licensing	7	10.9
R&D	7	4.6
Technology transfer	7	3.6
Drug development	6	10.2
Open source	6	9.5
Patents	6	9.5
Absorptive capacity	5	8.0
Business model	5	6.8
Entrepreneurship	5	29.4

## Discussion

In this work, we perform for the first time a full-scale bibliometric analysis of the literature on open innovation implications in medical and pharmaceutical research.

We found that publications that documented open innovation in medical and pharmaceutical research mainly began to appear in the mid-2000s. Many pharmaceutical and biotechnology companies were active in open innovation research, as the industry could provide consultations, contract research outsourcing, sponsored grants, and training and education and help spinout companies (Chin-Dusting et al., 2005). An interesting finding is that industry-affiliated articles were cited more often than nonindustry publications. This finding echoed the results of Narin and Rozek (1988), in which pharmaceutical industry articles were found to have higher mean citation counts than those of non-National Institutes of Health (NIH) publications but less mean citation counts than those NIH-supported articles. Generally, this could be interpreted as the research outputs of the industry-affiliated publications were of better quality; however, one of the potential reasons for the higher citation counts could be that the biotechnology/pharmaceutical companies have well-established networks and connections to disseminate research outputs to the academic audience and the general public. Moreover, compared to academic institutions, companies might invest more funding in visibility-promotion campaigns. An extensive analysis of marketing, governmental, and academic data has revealed that medical marketing expenditure in the United States increased from 18 to 30 billion from 1997 to 2016 (Schwartz and Woloshin, 2019). Indeed, findings from the current study demonstrate that industry articles also received more attention from online social media and thus had a higher Altmetric score. Readers should be aware that the Altmetric score is based on mentions in multiple social media channels such as Facebook and Twitter, as well as in Wikipedia, online blogs, and news outlets. The score does not reflect the context of the mentions. Along this line, a previous study showed that press releases by pharmaceutical companies tended to focus on qualitative results and harms and adverse events, but not on quantitative results and study limitations, which might create some bias of online attention to this research (Kuriya et al., 2008).

Besides, the predominant contributions from North America and Europe made this research field distinct from other biomedical fields such as neuropharmacology or ethnopharmacology, in which Asian and South American countries also had considerable inputs (Yeung et al., 2018; Yeung et al., 2020). Furthermore, in respect to the geographical distribution of author affiliations, the research literature on open innovation in medical and pharmaceutical research also differs from the literature on business incubators, in which China ranked 2^nd^ in terms of the number of published articles (Albort-Morant and Ribeiro-Soriano, 2016). Italy not only was prolific in terms of publication numbers but also produced highly cited articles, for example, an exploratory analysis on the top 20 worldwide biopharmaceutical firms that revealed the usage of various methods (licensing agreements, nonequity alliance, and purchase and supply of technical and scientific services) in partnering with other companies and universities for both inbound and outbound technologies and knowledge (Bianchi et al., 2011). Another highly cited article described the collaboration between the United States and Europe to establish a public-private partnership, called Open PHACTS, to facilitate drug discovery (Williams et al., 2012). A similar public-private partnership was reported in the United States that attempted to establish a national program of active drug safety surveillance by pooling observational data (Stang et al., 2010). In Europe, a BIGCHEM project, funded by the European Union (Horizon 2020), was initiated to foster big data analysis in the age of chemoinformatics to drive pharmaceutical research collaboration between the industry and academia (Tetko et al., 2016). With respect to Asia, open innovation research works were also published but gathered fewer than 30 citations each. For instance, a Japanese study evaluated how much scientific knowledge the pharmaceutical industry has assimilated from universities and research institutes and found that such assimilation was associated with patent applications and patent efficiency (Saito and Sumikura, 2010). In China, it was reported that pharmaceutical firms are involved more actively in open innovation and intellectual property protection, catching up fast once they managed to establish research and development departments and started collaborations (Ren and Su, 2015).

The open innovation movement has yielded a number of promising outcomes in drug discovery. One example is the identification of arylpyrroles with highly potent antimalarial properties through a broad industry-academia partnering and an open-source research approach involving real-time sharing of all data and ideas and enabling broad participation, without pursuing patents (Williamson et al., 2016). Moreover, it was found that JQ1, a thienotriazolodiazepine and BET inhibitor reported with its intellectual property released to the scientific community on an open access basis upon discovery, had a greater uptake by more interdisciplinary research communities and a larger number of downstream patents compared to the conventionally developed equivalents (Arshad et al., 2016).

Seventeen articles within the analyzed dataset were identified by searching for crowdsourc* to reveal crowdsourcing-related research applications. Though these articles were mainly about drug discovery and pharmaceuticals, examples from other sectors could also be identified. In the context of health and astronomy, the NASA Human Health and Performance Directorate organized crowdsourcing competitions to identify viable potential collaborations with the private sector in response to steep budget reductions (Davis et al., 2015). In medicine, contest-based crowdsourcing has similarly benefited the genome study community by identifying new approaches to accelerate the analysis speed of a standard genetics software package by 18–45-fold (Hill et al., 2017). In the context of health and food industry, crowdsourcing could be helpful by utilizing the crowd’s data in various safety and quality aspects such as developing systems of foodborne illness surveillance and identification of contaminated products (Soon and Saguy, 2017).

While our analysis reveals that open innovation is very common in the area of pharmaceutical development (Table 2), it can be observed that open innovation was also applied to other areas of the medical sector, such as medical devices. For example, a pair of open-source ptosis crutch glasses was invented to support the drooping eyelids of patients with myasthenia gravis, and the open-source platform approach allows further open innovation to improve the product (Saidi et al., 2018). Another example was a balloon sinuplasty device invented to treat severe chronic sinusitis. Upon observing the traumatic procedures of the conventional surgical approach, a medical device company invented a device to compress and displace structures in the congested area instead of cutting and removing; by collaborating with the academia to test and fine-tune the device, large-scale clinical studies finally proved its efficacy (Wan and Quan, 2014).

Electronic health records could also be optimized by open innovation in a bottom-up approach, by gathering the feedback of participants attending a workshop who worked together to identify shortcomings and propose solutions to streamline the steps in the workflow (Murphy et al., 2020). To promote the building of infrastructure for digital health, some open-source meta-data registry framework and core catalogs could be shared with the community to encourage collaborations and cross-disciplinary deployment (Hussey et al., 2019). Taking into consideration that information technologies were historically one of the pioneer areas adopting open innovation, as well as the recent development boost in sectors such as telehealth, artificial intelligence, virtual reality, and wearable technologies, it is very likely that in the future, there will be an increasing application of open innovation strategies in areas such as digital therapeutics and digital health technologies.

Interestingly, only two publications within the analyzed literature set referred to "citizen science", with one of them representing a review of Virtual Health Networks (Trequattrini et al., 2015) and the other one focusing on policy implications of open science (Stodden, 2010). Another aspect waiting to be further developed in the future is patient-centered innovation. Focus interviews have shown that stakeholders in the healthcare sector were keen on patient-centered care innovations (Reed et al., 2012), and initiatives have been launched in drug development (Hirsch, 2019) and diabetes therapy (O'Donnell et al., 2019). More patient-centered initiatives should be encouraged, and their efficacy evaluated in the future.

## Limitations

Our work focused on the bibliometric analysis of publication- and citation-related data and the semantic content of the title, abstract, and keywords. Therefore, systematic review or analysis of the product approval and improvements of health resulting from open innovation was not covered. Along this line, it should be kept in mind that the ultimate goal of innovation is to yield new products and, therefore, a detailed analysis of product approvals resulting from open innovation represents a very important direction for future work.

Since the search string used in our work included the metaword pharma*, it was expected that the research area of “pharmacology and pharmacy” would be highly ranked in the analyzed literature set. Therefore, readers should be aware that generalizations for the relative prevalence of open innovation in different research sectors should not be directly drawn based on the results of our study and, clearly, open innovation plays a very important role in other research areas outside the medical/healthcare/pharma sectors.

Since our study is based on data derived from just one database (Web of Science), there is an implication that some relevant articles might have been missed. However, different databases count citations differently, so it was not feasible to merge data from multiple databases for the present analysis.

## Conclusion

By studying 384 published research articles in the existing academic literature, it was found that open innovation in medical and pharmaceutical research began to appear in the literature in mid-2000s and most of the research was done in North America and Europe, with Asia lagging behind. Publications with authors affiliated with biotechnology or pharmaceutical companies had a higher mean citation count than nonindustry-affiliated articles and also received more social media attention. Various public-private partnerships that concerned financial and expertise support, sharing of intellectual property, and open platforms enabling easier communications, were introduced in the analyzed publications. Pharmaceuticals/pharmacology was revealed as an active industrial sector with medical significance promoting open innovation. However, taking into consideration the strong history of open innovation in the area of information technologies, it is feasible to expect that, in the future, there will be an increasing application of open innovation strategies from companies operating on the interface of information technology and medicine (e.g., in the area of digital health technologies). To harness the significant opportunities offered by open innovation, all relevant parties must be open to the changing landscape and embrace new models of collaboration with enthusiasm, without losing sight that the real quest is the development of solutions and products for the betterment of human health.

## Data Availability Statement

The original contributions presented in the study are included in the article/Supplementary Material; further inquiries can be directed to the corresponding authors.

## Author Contributions

AA and HW designed the study. AY and AA collected and analyzed data. AY and AA wrote the first draft. HS, EK, FE, SV-K, MK-P, HW, and ES critically revised the draft. All authors agreed to the final version.

## Conflict of Interest

The authors declare that the research was conducted in the absence of any commercial or financial relationships that could be construed as a potential conflict of interest.
